# Treatment of type III dens invaginatus in bilateral immature mandibular central incisors: a case report

**DOI:** 10.1186/s12903-022-02059-8

**Published:** 2022-02-04

**Authors:** Jie Zhang, Yuan Wang, Lei Xu, Zhifang Wu, Yan Tu

**Affiliations:** Stomatology Hospital, School of Stomatology, Zhejiang University School of Medicine, Zhejiang Provincial Clinical Research Center for Oral Diseases, Key Laboratory of Oral Biomedical Research of Zhejiang Province, Cancer Center of Zhejiang University, Hangzhou, 310006 China

**Keywords:** Dens invaginatus, Apical periodontitis, Endodontic treatment, Immature permanent teeth

## Abstract

**Background:**

Dens invaginatus (DI) is a developmental anomaly, Oehlers Type III DI is the most complex type and early diagnosis and treatment is complicated and challenging. This report presents a rare case of a type IIIb DI associated with a periapical lesion in bilateral immature permanent mandibular central incisors.

**Case presentation:**

An eight-year-old boy referred to our clinic manifesting with pain along with swelling in the mandibular incisors for the past one month. Radiographic examination showed periapical radiolucency exhibiting a scantly defined border, as well as an invagination which had a central invaginated canal extending from the pulp chamber throughout the apical foramen in both mandibular central incisors. We performed two different treatment procedures on the basis of the condition of the main pulp of the mandibular central incisors. in which only the invagination root canal was treated in the right mandibular central incisor, while the invagination and main root canals were treated in the left mandibular central incisor. During the 18-month follow-up period, the teeth were clinically asymptomatic. Imaging examinations indicated complete healing of the periapical lesion and revealed that the wall of the root canal was thickened and the open apex was closed.

**Conclusion:**

For young permanent teeth with type III Dens invaginatus, it is particularly important to keep pulp vitality, which could maintain root development and allow a good long-term prognosis.

## Background

Dens invaginatus (DI), usually referred to as “dens in dente,” is an uncommon developmental anomaly that causes invagination or deepening of the enamel organ into the dental papilla before the mineralization stage [1]. The precise etiology of DI remained indistinct but it tends to include genetic along with environmental factors. Numerous theories have been opined, including infection, trauma, changes in tissue pressure, or local discrepancies in cellular hyperplasia [2]. Nevertheless, the exact etiology is still unclear.

Dens invaginatus is a considerably common condition with an estimated incidence of between 0.3% and 10% in all teeth [3], with maxillary lateral incisors being the most usually affected, and subsequently by the maxillary central incisors, but it is rare in the mandibular incisor [4].

Several classification systems for dens invaginatus have been proposed, including dens in dentes, dentoids in dentes, invaginated odontomes, and dilated composite odontomas [5], but the most widely used and clinically relevant classification system was proposed by Oehlers [6]. According to the depth of enamel invagination observed radiographically, this classification system divides invagination into three types. In type I, there is minimal invagination that is limited to the crown; in type II, the invagination extends beyond the cementoenamel junction (CEJ) in the inform of a blind sac. Type IIIa, the invagination invades throughout the root and laterally extends to the periodontal tissues. In type IIIb, the invagination penetrates through the root and apically opens in the periapical tissues but does not communicate with the pulp. An infection in a type III invagination could result in an inflammatory response within the periodontium and periapical tissues, leading to periapical periodontitis, unrelated of pulp vitality [7, 8].

It is usually feasible for only “endodontic treatment” of the invagination to keep the main root canal’s pulp vitality, especially if the tooth is an immature permanent tooth with vital pulp, whereas the invagination possesses a separate apical or lateral foramen. The goal of the therapy could be to keep pulp vitality of the tooth for additional root development, although there are extensive management methods for these cases [9].

This case report describes a rare case of type IIIb DI linked to a periapical lesion in bilateral immature permanent mandibular central incisors, in which only the invagination root canal was treated in the right mandibular central incisor, on the contrary the invagination and main root canals were treated in the left mandibular central incisor. Ultimately, the two teeth had a favorable prognosis. During the 18-month follow-up period, sufficient healing in the soft tissue, as well as the periapical lesion and continuous root development of the teeth were observed. Furthermore, pulp vitality of the right mandibular central incisor was maintained in the main canal.

## Case presentation

**Fig. 1:**
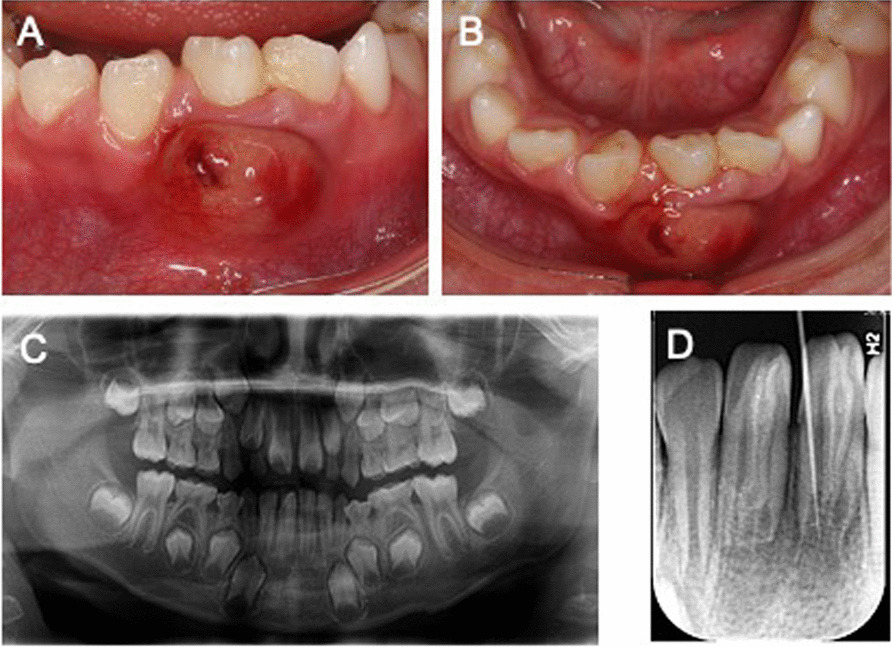
Preoperative clinical view and radiographs of the dens invaginatus (#31and #41): Clinical view shows that the bilateral mandibular central incisors were under eruption with coronal anomaly. (**A** and **B**; labial and incisal views, respectively). **C** The mandibular dental arch panoramic radiograph including the normal number of teeth and anatomical aberration (#31and #41). **D** Periapical radiograph showing a type III DI extending from the pulp chamber throughout to the apical foramen linked to periapical radiolucency and incomplete root formation

An eight-year-old boy referred to our clinic manifested with pain along with swelling in mandibular incisors for the past one month, especially the past week. No relevant medical, family or psychosocial history was reported. There was no abnormality on extraoral examination. An abnormal coronal anatomy of the bilateral mandibular central incisors was found on intraoral examination. The conical crown had a deep depression with a talon cusp on the conical crown, and caries or restorations were not found in the teeth. There was a giant abscess on the labial gingiva of the left mandibular central incisor, which exhibited pain on vertical percussion and degree II tooth mobility. Concurrently, there was a fistula on the distal and lingual gum of the right mandibular central incisor, which exhibited slightly pain on vertical percussion and degree-I tooth mobility (Fig. 1A, B).

**Fig. 2:**
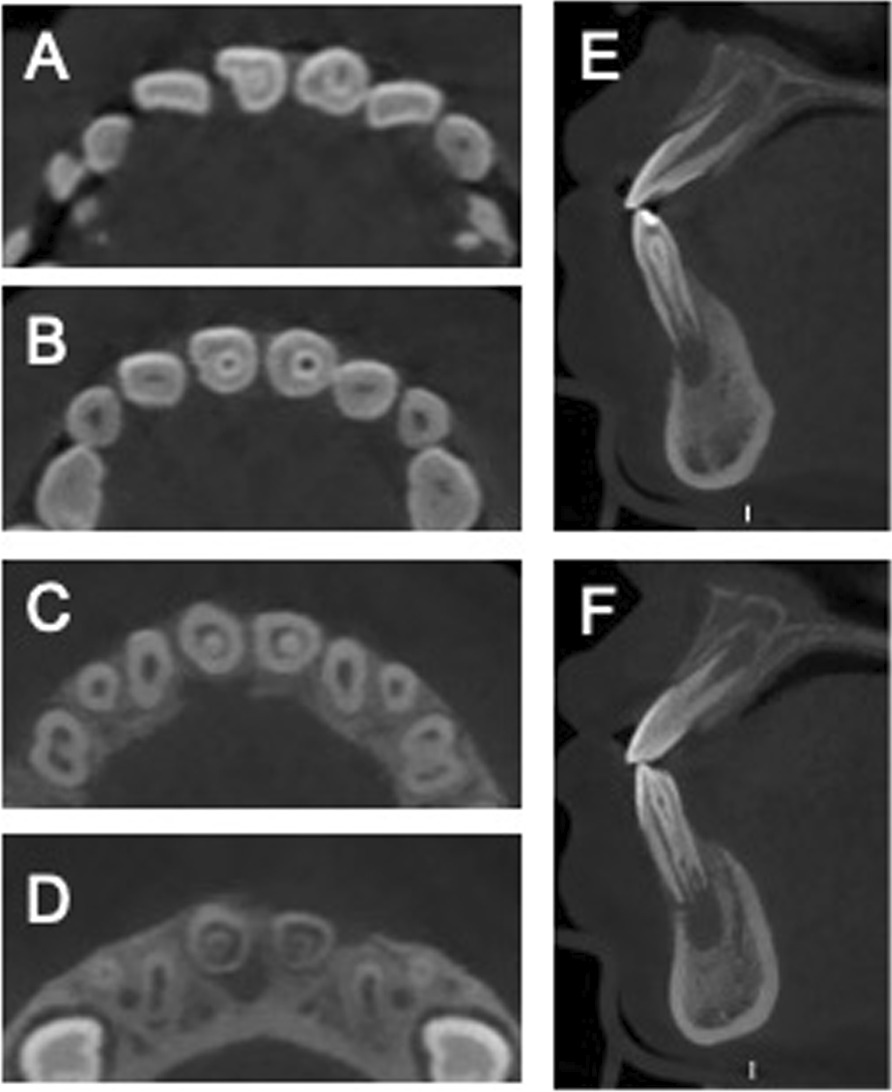
The preoperative CBCT images of the dens invaginatus. The axial sections from coronal to apical are illustrated in **A**–**D**, the sagittal views illustrating the DI with the periapical lesion (E #31 and F #41)

Radiographic examination showed periapical radiolucency exhibiting a scantly defined border, as well as an invagination which had a central invaginated canal extending from the pulp chamber throughout the apical foramen in the mandibular central incisors (Fig. 1C, D). This type of anatomy was consistent with a DI Odhlers type IIIb. Although there was no response on the electric pulp vitality test, there were doubts about the dependability of this result due to immature development of the root with an open apex. We performed a CBCT (cone‑beam computed tomography) scan as a complementary examination to acquire more comprehensive anatomic information and an accurate diagnosis. The CBCT images exhibited an invagination of the bilateral mandibular central incisors extending from the crown throughout to the root canal apex; however there was no communication with the main root canal. The apical foramen was incomplete and had a large area of periapical radiolucency of approximately 5.2 × 7.8 mm in the left mandibular central incisor and approximately 4.1 × 6.4 mm in the right mandibular central incisor (Fig. 2A–H).

We diagnosed the case as DI with chronic apical periodontitis. Extraction and orthodontic intervention are important because of the complex root involvement and the uncertain prognosis of conservative therapy. Nevertheless, the patient’s parents hoped to retain the natural teeth. Endodontic therapy was selected as the preferred treatment plan.

According to the radiographic examination, we found that there was no communication between the invagination and the main root canal. It was postulated that the pain and infection were caused by infection in the invagination, while the main root canal pulp remained vital. Under rubber dam isolation and a dental surgical microscope (Leica M330; Leica microsystems, Wetzlar, Germany), conservative endodontic access into the invagination was made using a small round bur, while the main root canal pulp was not exposed. The orifice of the invagination was confirmed with an endodontic explorer and 15# K-file. No penetration was observed between the invaginated canal and the main root canal. At the same time, the periapical tissues discharged a bloody and purulent exudate. Stainless-steel hand K-files along with nickel-titanium rotary instruments were employed to instrument the invaginated canal; however, with caution not to surpass the length of the canal. In the process, the invagination was irrigated thoroughly with 1.5% sodium hypochlorite solution (NaOCl) and 0.9% saline solution. Moreover, a sonic-activated device was employed to attain a more effective debridement. It was necessary to irrigate the invagination thoroughly due to the complex canal structure. Paper points were employed to dry the invaginated canal, followed by application of paste of calcium hydroxide, and then temporary filling material (Caviton; GC Co., Tokyo, Japan) was employed to seal the access cavity.

**Fig. 3:**
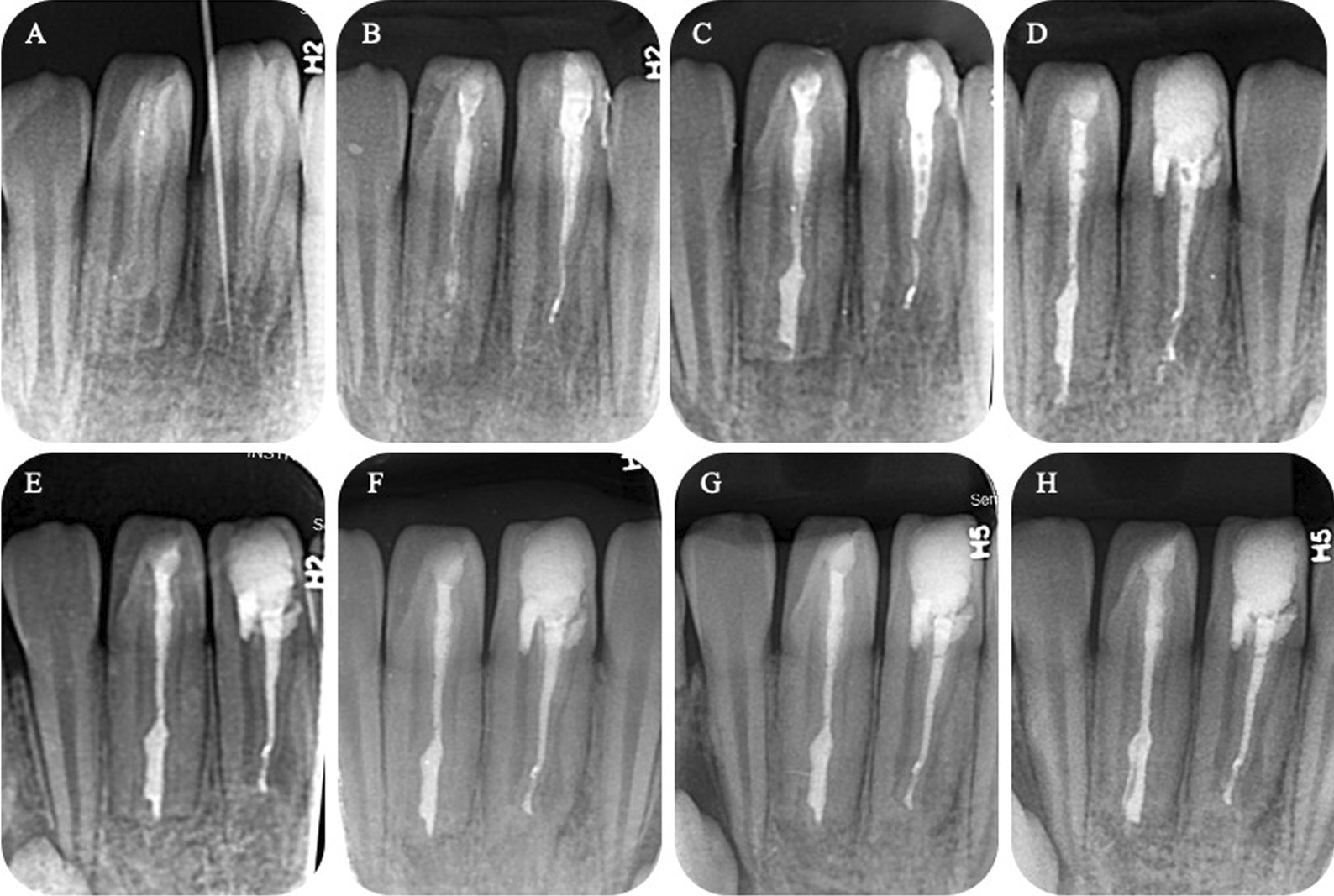
Treatment and follow-ups periapical radiographs. **A** preoperative, **B** postoperative after the first vitapex filling, **C** postoperative after the second vitapex filling, **D** postoperative after iRoot BP plus filling of the main root canal and iRoot SP and warm gutta-percha obturation of the invaginated root canal (#31), and at **E** 5-month, **F** 1-year, and **G** 1.5-year recall. The radiographic image indicates apex closure of the main root canal and root development of the tooth. **H** Postoperative after iRoot SP and warm gutta-percha obturation of the invaginated root canal (#41)

During the second appointment after 2 weeks, the patient felt well, and the fistula on the distal and lingual gum of the right mandibular central incisor had disappeared. The giant abscess on the labial gingiva of the left mandibular central incisor was relieved but not healed. Abundant irrigation using 17% ethylenediaminetetraacetic acid (EDTA) solution was employed to remove the intracanal dressing of the invagination along with the smear layer. Subsequently, copious 1.5% NaOCl and saline with the sonic-activated device were applied. After that, paper points were employed to dry the invaginated canal, which was then obturated with the Vitapex (Neo dental co., Tokyo, Japan). Then we took the radiographic imaging of the teeth to confirm the position of the Vitapex (Fig. 3B), and GIC (glass ionomer cement) (GlasIonomer FX-II; Shofu Inc, Kyoto, Japan) was used to seal the access cavity.

At the third appointment after 1 month, the patient felt well, and the right mandibular central incisor had no clinical signs or symptoms. There was still an abscess on the labial gingiva of the left mandibular central incisor, although it was smaller. For the right mandibular central incisor, Vitapex was obturated in the invagination canal to induce root development, and then used GIC and composite resin (Z350; 3M ESPE, St Paul, MN, USA) to seal the access cavity. For the left mandibular central incisor, it was identified that main root canal pulp was infected, and treatment was required. Under local anesthesia utilizing articaine with 1:10,000 epinephrine, small round bur was employed to prepare an endodontic access to the main root canal, and the pulp was found to be necrotic. A stainless-steel hand K-files was employed to simply instrument the main root canal and was irrigated abundantly with 1.5% NaOCl and 0.9% saline solution with a sonic-activated device to obtain more effective chemical debridement. Paper points were used to dry the main root canal, followed by application of calcium hydroxide paste via syringe, and the access cavity was sealed with GIC. At the same time, we replaced the dressing in the invagination with Vitapex (Fig. 3C).

At the fourth appointment after three weeks, there were no clinical signs or symptoms of the left mandibular central incisor. 2% mepivacaine hydrochloride (no epinephrine) was used to perform local anesthesia, followed by reaccession of the main root canal. Next, 0.9% saline along with a sonic-activated device were utilized to remove the calcium hydroxide paste. Gentle irrigation of the canal with 17% EDTA solution (20 mL) was performed and followed by drying using paper points. The canal was over-instrumented with a precurved K-file extending 2 mm past the apical foramen, to induce bleeding. The instrument was extended 3–4 mm below the CEJ (cementoenamel junction) to allow formation of a bold clot. We placed an iRoot BP Plus (Innovative BioCeramix, Vancouver, BC, Canada) on top of the blood clot. At the same time, iRoot SP (Innovative BioCeramix) and warm gutta-percha obturation replaced the Vitapex in the invaginated root canal. Then, the tooth was sealed with GIC and was restored using a composite resin. A radiographic image verified the position of the iRoot BP Plus (Fig. 3D).

Eighteen months later, there were no clinical signs or symptoms of the mandibular central incisors. According to the radiographic image, the open apex was closed, and the root continued to develop. Therefore, iRoot SP and warm gutta-percha obturation replaced the Vitapex in the invaginated root canal of the right mandibular central incisor, and then the tooth was finally restored with composite resin (Fig. 3G–H).

## Discussion and conclusions

Dens invaginatus is a maldevelopment of the tooth, which occurs because of enamel or dentin folding into the cavity and the root [10]. These structural defects of DI are predisposing factors of caries as a result of the deep pits resulting in the onset of caries. In particular, microorganisms along with their products could exacerbate infection and ultimately result in pulp necrosiss [11]. Regarding dens invaginatus type III, which is the most serious type and has a more complicated root canal system, early diagnosis accompanied with treatment of DI is needed.

Treatment of teeth with serious type III DI penetrating to the apex with a periapical lesion is always complex, with many challenges. As to severe infection exists, some treatment approaches may be preferred, including root canal treatment, replantation, surgery, and even extraction [12]. There is no doubt that type III DI is an endodontic treatment challenge on account of the complicated morphology of root canal and the hardship in accessing irregular, as well as invaginated canals [4, 13]. Three-dimensional imaging formed by CBCT is essential in diagnosing and managing DI because it can provide comprehensive information regarding the internal morphology, as well as guide treatment [13, 14].

For treatment planning, an accurate evaluation of the status of the main pulp is critical. If the main pulp is vital, it is likely to maintain pulp vitality of the main canal by cleaning along with filling of the invaginated canal. Lots of reports have proven the success of this treatment [15]. If the main canal, as well as the invaginated canal are infected, it is necessary to clear them respectively and fill them densely [15, 16]. Especially, when the main canal is immature, apexification and regenerative endodontic treatment of the main canal is recommend [13, 17].

Regarding type IIIb DI, some dentists will remove all the invagination tissues for improved disinfection, as well as instrumentation of the root canal system [18]. The option of removing the invagination should be carefully considered since the process is very difficult, complex and not appropriate for all cases. Surgery may be is needed, when conservative therapy fails or when the invaginated canal cannot be completely cleaned and filled using traditional approaches [14, 19]. It is a challenge to clean and shape the invaginated canal because of the irregular shape of the root canal system; therefore, engine-driven rotary nickel-titanium instruments should be utilized very cautiously during treatment [19]. In addition, it is necessary to use copious NaOCl irrigation combined with a sonic-activated device because some areas may be completely inaccessible by instrumentation. Once root canal preparation is performed, it is generally acceptable to use gutta-percha for the filling procedure. nevertheless, when the apical anatomy of the root canal is complex and not suitable for conventional filling, bio-ceramics are better option for the filling material [20, 21].

This case report shows a rare case of type IIIb DI related to a periapical lesion in bilateral immature permanent mandibular central incisors. In case of immature permanent teeth, it is particularly significant to keep pulp vitality, which could allow uninterrupted root development and alow a good long-term prognosis, with full function facilitated by a mature structure and vitality. In the present case, bilateral immature permanent mandibular central incisors had clinical symptoms and periapical lesions but could be managed successfully. According to three-dimensional imaging performed by CBCT and clinical examination, the invagination was completely separated from the main root canal, so the invaginated canal was treated first. After the treatment, the right mandibular central incisor was healing successfully because only the invaginated canal was infected and the main pulp was still vital. In contrast, the left mandibular central incisor did not respond favorably because both the main canal along with the invaginated canal were infected. Therefore, we performed regenerative endodontic treatment of the main root canal because the main canal was immature with an open apex. Ultimately, the two teeth had a favorable prognosis. During the 18-month follow-up period, adequate healing of the soft tissue, as well as the periapical lesion and radiographic image revealing apex closure and root development were obtained. Furthermore, we used the electric pulp vitality tester to test the pulp vitality of two teeth, weak pulp vitality was maintained in the right mandibular central incisor.

Endodontic therapy for teeth with type III DI that penetrates to the apical area and is combined with a large, symptomatic periapical lesion is very challenging. The principal consideration for selecting treatment methods is maintaining pulp vitality and long‑term prognosis or preserving the tooth via the least invasive method. Therefore, complicated methods that need accurate diagnosis along with proper treatment planning are generally involved. Three-dimensional imaging performed by CBCT provided appropriate details on the anatomy, and therapy using a dental surgical microscope and bio-ceramics was critical to allow predictable, as well as successful results [2].

## Data Availability

All data generated or analysed which related this case report are included in this published article.

## Abbreviations

CEJ: Cementoenamel junction
CBCT: Cone-beam computed tomography
DI: Dens invaginatus
EDTA: Ethylenediaminetetraacetic acid
GIC: Glass ionomer cement
NaOCl: Sodium hypochlorite solution
